# Platelet-Derived Growth Factors Affect Clinical Features and Prognosis of Gastric Cancer

**DOI:** 10.1155/2022/2108368

**Published:** 2022-08-18

**Authors:** Xia Zhao, Zhihao Yu, Ku Zang

**Affiliations:** ^1^Department of Ultrasound Medicine, The Affiliated Huaian No. 1 People's Hospital of Nanjing Medical University, Huaian 223300, China; ^2^Department of Intensive Care Unit, The Affiliated Huaian No. 1 People's Hospital of Nanjing Medical University, Huaian 223300, China

## Abstract

**Purpose:**

To investigate the association of platelet-derived growth factors (PDGFs), clinicopathological features, and prognosis in gastric cancer patients.

**Methods:**

Tumor specimens of 180 individuals with gastric cancer treated between 2016 and 2020 were collected. Immunohistochemical staining and Western blot (WB) were used to detect the expression of PDGF-B and PDGF-D. The relationship between the expression of PDGF-B and PDGF-D and relapse-free subsistence (RFS) time was assessed using Kaplan–Meier curves. Univariate and multivariate Cox proportional hazards regression models were used to evaluate the relationship between the expression of PDGF-B and PDGF-D and the prognosis and clinicopathological features in gastric cancer patients.

**Results:**

High expression of PDGF-B and PDGF-D was detected in 108 (60%) and 137 (76%) tumor specimens, respectively. The expressions of PDGF-B and PDGF-D were independent predictive indicators in multivariate analysis when compared to tumor depth, tumor stage, lymph node metastasis, and RFS (*P* < 0.01).

**Conclusion:**

The high expression of PDGF-B and PDGF-D in gastric cancer tissues is associated with poor prognosis and poor survival rate of the patients. The expression of PDGF-B and PDGF-D can be used as important indicators to evaluate the biological behavior and prognosis of gastric cancer.

## 1. Introduction

Gastric cancer (GC) is the most common gastrointestinal tumor worldwide and one of the leading causes of cancer-related death [1]. Early-stage gastric cancer has no particular signs or symptoms, which leads to delayed diagnosis and poor 5-year survival rate. For localized gastric cancer, complete surgical resection is the only curative therapy option. Patients with advanced gastric cancer, on other hand, have a poor prognosis. Despite radical resection, most patients with advanced gastric cancer will experience recurrence or metastasis [2]. Even with intensive chemotherapy, patients with recurrence or metastasis have a median survival period of less than 13 months [3]. Thus, developing novel molecular markers and therapeutic targets for gastric cancer is critical for improving therapy and prognosis of the disease.

The prognosis of gastrointestinal tumor is associated with their clinicopathological features. In addition to factors such as age, general health, and response to treatment, the type of cancer and its location, stage, and grade affect the prognosis of gastric cancer. Gastric cancer contains aberrant expression of numerous cytokines that are linked to the grade, stage, and prognosis. Abnormal cytokines are involved in the occurrence and development of gastric cancer, and regulating their expression levels may become a new approach of treatment for gastric cancer [4]. Elevated receptor tyrosine kinases, such as epidermal growing factor receptor (EGFR), fibroblast growth factor 2 (FGF2), c-Kit, platelet-derived growth factor (PDGF), and its receptor (PDGFR), have been shown to affect the prognosis of gastric cancer patients. In addition, PDGFs and PDGFRs have been linked to the onset of gastric cancer.

In 1987, PDGF was extracted from human blood vessels as a pro-angiogenic factor. It is a common peptide regulator that promotes connective tissue development, which helps with tissue healing, immunological response, tumor cell proliferation, and other functions. The four subtypes of PDGF are PDGF-A, PDGF-B, PDGF-C, and PDGF-D. PDGF-B, PDGF-C, and PDGF-D, for example, are increased in various malignancies and play key roles in tumor growth, angiogenesis, and metastasis [5, 6]. Suzuki et al. discovered that PDGF-B is critical in angiogenesis in intestinal-type gastric cancer.

Treatment that targets the PDGF-B signaling pathway was successful [7]. This study intends to investigate the relationship among the expression of PDGF-B and PDGF-D, clinicopathological features, and prognosis in patients with gastric cancer and to provide experimental and theoretical basis for clinical diagnosis, treatment, metastasis prediction, and prognosis evaluation of gastric cancer.

## 2. Materials and Methods

### 2.1. Sample Collection

A retrospective analysis was performed on 180 patients who received curative gastrectomy in the Department of Esophagogastric Surgery at the Affiliated Huai'an No.1 People's Hospital of Nanjing Medical University between January 2007 and December 2011. The average age of patients was 65.34 years (26–85 years). Inclusion criteria were as follows: (1) gastric adenocarcinoma diagnosed by pathology; (2) the patient did not receive radiotherapy, chemotherapy, immunotherapy, and targeted therapy before surgery; and (3) the patient did not have serious cerebrovascular and metabolic diseases. Exclusion criteria were as follows: (1) patients with incomplete clinical or follow-up data and (2) patients with multiple primary tumors. The patient clinical characteristics are summarized in Table 1. The International Union for Cancer Control [8] recommends using the tumor-lymphnode-metastasis categorization criteria for each tumor. Diagnostic imaging, such as computed tomography, ultrasonography, and endoscopy, was used in patients every 3–6 months to check for recurrent illness. This study was approved by the Institutional Review Committee of Huai'an First Hospital affiliated to Nanjing Medical University, and written informed consent was obtained from all patients.

### 2.2. Immunohistochemical Staining

Immunohistochemical staining was performed using the Histofine Simple Stain MAX PO (MULTI) method. 4 *µ*m thick sections were cut from formalin-fixed, paraffin-embedded tissue blocks. After deparaffinization and rehydration in graded ethanol, slides were treated with 3% hydrogen peroxide for 15 min. Slides were incubated overnight at 4°C with primary antibodies against PDGF-B (diluted, 1 : 50) or PDGF-D (diluted, 1 : 50). Sections were incubated with peroxidase-conjugatedanti-goat or anti-rabbit antibodies for 30 min at room temperature. Peroxidase activity was detected with diaminobenzidine. Slides were counterstained with 1% Mayer's hematoxylin. Staining intensity was rated on a four-point scale: 0 (none), 1 (weakly positive), 2 (moderately positive), and 3 (highly positive). Grade regarding stain was categorized into four grades: 0, <25.0%; 1, 25.0% to <50.0%; 2, 50.0% to <75.0%; or 3.0, ≥75.0%. The composite score is calculated by adding the intensity score to the degree score. For statistical analysis, composite scores ≥4 were defined as high expression, and scores <4 were considered low expression. Normal tissues from the same patients were used as controls. In negative controls, antibodies were replaced with normal goat or rabbit IgG.

### 2.3. Statistical Analysis

SPSS 22.0 statistical analysis software was used for statistical analysis. The connection between PDGF-B and PDGF-D expression and recurrence-free survival (RFS) time was measured by means of Kaplan–Meier curves and log-rank examination. *P* < 0.05 was considered statistically significant. The prognostic significance of PDGF-B and PDGF-D expression as well as clinicopathological variables was measured by means of univariate and multivariate Cox regression analysis.

## 3. Result

### 3.1. Immunohistochemical Staining

**Figure 1:**
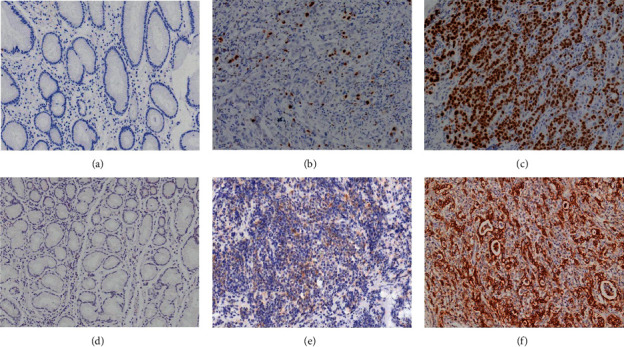
PDGF-B and PDGF-D immunostaining. (a) PDGF-B negative staining (100 × 100). (b) PDGF-B weak staining (100 × 100). (c) PDGF-B strong positive staining (100 × 100). (d) PDGF-D negative staining (100 × 100). (e) PDGF-D weak staining (100 × 100). (f) PDGF-D strong positive staining.

In this study, high expression of cellular PDGF-B and PDGF-D was detected in 108 (60%) and 137 (76%) samples, respectively. PDGF-B and PDGF-D expressions were found in 88 (48.89%) tumor tissues, whereas low expression of both PDGF-B and PDGF-D was seen in 36 (20.00%) tumor tissues. In addition, we observed the expression of PDGF-B and PDGF-D in 79 metastasis-positive lymph nodes. In these samples, 81.01% of samples had high PDGF-B expression and 87.34% had high PDGF-D expression.  Figure 1 shows the immunostaining patterns of PDGF-B and PDGF-D. There was no significant difference in the expressions of PDGF-B and PDGF-D between primary tumors and metastatic lymph nodes (*P* > 0.05), as shown in Table 2.

### 3.2. Analysis of the Relationship between Clinicopathological Parameters and Expression of PDGF-B and PDGF-D

As indicated in Table 3, differentiated tumors had higher PDGF-B and PDGF-D expression than undifferentiated tumors (*P* < 0.01). The high expression of PDGF-B and PDGF-D was significantly correlated with tumor depth, tumor stage, and lymph node metastasis (*P* < 0.01). Age and gender differences were not significant (*P* > 0.05).

### 3.3. The Relationship between the Expression of PDGF-B and PDGF-D and RFS

**Figure 2:**
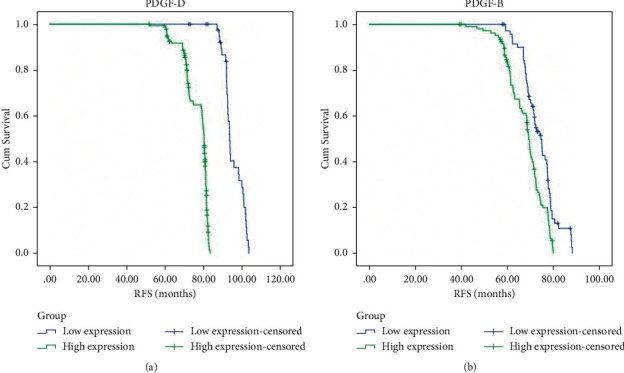
Kaplan–Meier curve of recurrence-free survival (RFS) in patients expressing PDGF-D (a) and PDGF-B (b).

Compared with the low PDGF-D expression group, the RFS of the PDGF-D high expression group was significantly shortened (93.51 ± 10.25 months vs 80.23 ± 9.18 months, *P* < 0.01) (Figure 2(a)). Likewise, compared with the PDGF-B low expression group, the RFS of the PDGF-B high expression group was significantly shortened (74.44 ± 6.91 months vs 69.10 ± 6.22 months, *P* < 0.01) (Figure 2(b)).

### 3.4. Univariate and Multivariate Cox Analysis of the Survival Time of Gastric Cancer Patients

Histopathology, tumor depth, lymph node metastasis, and high expression of PDGF-B and PDGF-D (all *P* < 0.01) were variables impacting the survival time of gastric cancer patients in a Cox regression analysis. As indicated in Table 4, high PDGF-B and PDGF-D expression, histology, tumor depth, and lymph node metastasis were all independent factors for gastric cancer patient survival.

## 4. Discussion

Gastric cancer is the most common malignant tumor in China. Antitumor drug therapy is one of the main treatments for unresectable advanced gastric cancer. From traditional cytotoxic drugs to precise molecular targeted drugs and immune checkpoint inhibitors, patients with advanced gastric cancer have more and more drug options. With advanced surgical approaches combined with improved chemotherapy, the survival rate of gastric cancer patients, especially early gastric cancer patients, has been greatly improved. However, patients with advanced gastric cancer have a mortality between 25% and 30%, and their prognosis is poor [8]. Early detection, accurate staging, and continuous monitoring are required for effective treatment of gastric tumor. However, there are no biomarkers that can accurately predict prognosis of gastric cancer. Exploring new biomarkers to improve the prognosis of gastric cancer has significant clinical implications [9].

Platelets, fibroblasts, endothelial cells, smooth muscle cells, and osteosarcoma cells remain all sources of PDGF. High expression of PDGF-B and PDGF-D in this research was shown to be strongly connected with tumor depth, tumor stage, and lymph node metastasis. PDGF-D was first discovered in 2001. It is a chemokine of vascular smooth muscle. By inducing the formation of tumor blood vessels, it promotes proliferation of tumor cells and participates in the occurrence and development of many malignant tumors [10]. The platelet-derived growth factor receptor (PDGFR) is a member of the tyrosine protein kinase family that is found on the cell membrane and in a variety of stromal cells. Most malignancies have increased PDGFR expression [11]. PDGF-D may attach to its receptor PDGFR-*β*, leading to phosphorylation to control tyrosine kinase, which allows its actions in cells [12, 13]. PDGF-D is frequently upregulated in various types of cancer and plays an important role in tumor progression, angiogenesis, and metastasis through multiple oncogenic pathways, including phosphatidylinositol 3-kinase/Akt, nuclear factor-*κ*B (NF-*κ*B), extracellular signal-regulated kinases, mitogen-activated protein kinases, and the Notch pathway [14, 15]. Furthermore, PDGF-D may be linked to epithelial-mesenchymal transition (EMT), a critical step in tumor metastasis, through multiple signaling pathways such as Notch and NF-B. High PDGF-D expression was shown to be related to EMT in prostate cells by Kong et al. [16]. Zhao et al. [17] also found that using RNA interference to silence PDGF-D dramatically abridged the propagation and invasion of gastric cancer cells overexpressing PDGF-D. In this research, PDGF-D overexpression was seen in 76% of gastric cancer patients, representing that blocking PDGF-D might be a useful treatment approach.

The proto-oncogenec-sis encodes the protein PDGF-B. It may enhance the production of tumor vascular endothelial cells and development of new blood vessels, as well as tumor growth, invasion, and metastasis, as a key angiogenic factor [18]. PDGF-B may cause endothelial cell proliferation, passage, and the creation of tube-like vascular structures, according to Snao et al. [19]. Cao et al. [20] found that PDGF-B may increase lymph node metastasis of malignant tumors by inducing lymphatic vessel development and angiogenesis in tumors in a VEGF-independent way. PDGF-B is abundant in colon cancer tissues and has been linked to invasion, metastasis, and prognosis [18]. In this study, immunohistochemistry and WB experiments showed that PDGF-B expression in advanced gastric cancer specimens was higher than that in normal gastric mucosa specimens, and that it was higher in middle/advanced stage gastric cancer tissues than in early gastric cancer tissues, implying that PDGF-B plays a role in the occurrence and development of gastric cancer. Also, high PDGF-B expression is significantly associated with tumor complexity and lymph node metastasis, indicating that gastric cancer tissues with high PDGF-B expression may have high possibility of lymph node metastasis.

Although earlier studies have revealed that medication targeting the PDGF-B signaling pathway may be helpful in intestinal-type gastric cancer [7], this research was the first to use immunohistochemistry and a WB assay to identify PDGF-B and PDGF-D in gastric cancer. Univariate and Cox regression were used to examine the expression levels of subtype PDGF variables, as well as the association between clinicopathological markers and prognosis in gastric cancer patients. The findings revealed that high PDGF-B and PDGF-D expressions were meaningfully linked to tumor depth, tumor stage, and lymph node metastasis. High PDGF-D expression is significantly correlated with shorter RFS time, and high PDGF-D and PDGF-B expression, tumor depth, and lymph node metastasis were independent prognostic factors. The findings reveal that increased PDGF-B and PDGF-D expression in gastric cancer tissue is linked to the onset and development of gastric cancer, as well as poor prognosis and survival rate of gastric cancer patients. As a result, these parameters may be used to assess the biological behavior and prognosis of gastric cancer.

## Figures and Tables

**Table 1:** Clinical characteristics of study subjects (n (%)).

Characteristic	Value
Age, years	
<60	80 (44.44)
≥60	100 (55.55)

Gender	
Male	120 (66.67)
Female	60 (33.33)

Tumor location	
Higher third of stomach	28 (15.56)
Middle/lower third of stomach	152 (84.44)

Lymph node metastasis	
Optimistic	79 (43.89)
Damaging	101 (56.11)

Depth of invasion	
T1.0	45 (25.00)
T2.0	35 (19.44)
T3.0	50 (27.78)
T4.0	50 (27.78)

TNM phase	
I.0	108 (60.00)
II.0	45 (25.00)
III.0	27 (15.00)

**Table 2:** PDGF-C and PDGF-D expression in tumor and metastatic lymph node.

Primary tumor	Metastatic lymph nodes		*χ* ^*2*^	*P*
	Low	High		
PDGF-B			0.001	0.97
Low	5	21		
High	10	43		
PDGF-D			0.02	0.90
Low	2	15		
Tall	8	54		

**Table 3:** Clinicopathological features and expression of PDGF-B and PDGF-D.

Variables	PDGF-B expression		*χ* ^*2*^	*P*	PDGF-D expression		*χ* ^*2*^	*P*
	Low (*n* = 72)	High (*n* = 108)			Low (*n* = 43)	High (*n* = 137)		
Age, years			0.84	0.36			2.09	0.15
<60	35	45			15	65		
≥60	37	63			28	72		

Gender			1.67	0.20			1.85	0.17
Male	52	68			25	95		
Female	20	40			18	42		

Histopathology			15.18	<0.01			9.78	<0.01
Differentiated	24	68			15	85		
Undifferentiated	48	40			28	52		

Depth of invasion			17.78	<0.01			6.37	0.01
T1	30	15			17	28		
T2/T3/T4	42	93			26	109		

Lymph node metastasis			16.88	<0.01			15.36	<0.01
N0	45	34			30	49		
N1/N2/N3	27	74			13	88		

TNM stage			8.16	<0.01			27.89	<0.01
I	38	34			32	40		
II/III	34	74			11	97		

**Table 4:** Prognostic variables on a multivariate Cox regression analysis model.

Variables	Patients, n	Univariate analysis (*P* value)	Hazard ratio (95% confidence interval)	Multivariate analysis (*P* value)
Age, years		0.78		
<60	80			
≥60	100			

Gender		0.82		
Male	120			
Female	60			

Histopathology		<0.01	1.7 (1.0–3.0)	0.06
Differentiated	88			
Undifferentiated	92			

Depth of invasion		<0.01	1.8 (1.0–3.1)	0.04
T1	45			
T2/T3/T4	135			

Lymph node metastasis		<0.01	9.8 (2.3–36.2)	<0.01
N0	101			
N1/N2/N3	79			

PDGF-B expression		<0.01	4.7 (2.2–10.3)	<0.01
Low	72			
High	108			

PDGF-D expression		<0.01	3.2 (1.8–10.5)	<0.01
Low	43			
High	137			

## Data Availability

The data are available from the corresponding author upon request.
